# Multidisciplinary teams of case managers in the implementation of an innovative integrated services delivery for the elderly in France

**DOI:** 10.1186/1472-6963-14-159

**Published:** 2014-04-07

**Authors:** Matthieu de Stampa, Isabelle Vedel, Hélène Trouvé, Joël Ankri, Olivier Saint Jean, Dominique Somme

**Affiliations:** 1 Université Versailles St-Quentin, EA 2506 Laboratoire Santé-Environnement-Vieillissement, AP-HP, Hospitalisation à Domicile (HAD), 14 rue Vésale, 75005 Paris, France; 2 Department of Family Medicine, Lady Davis Institute, McGill University, Jewish General Hospital, Montreal, Quebec, Canada; 3 Fondation Nationale de Gérontologie, Paris, France; 4 EA 2506 Laboratoire Santé-Environnement-Vieillissement, Assistance Publique Hôpitaux de Paris, Hôpital Européen Georges Pompidou, Service de gériatrie, Paris, France; 5 Service de Gériatrie, Centre Hospitalier Universitaire de Rennes, Université de Rennes 1, Paris, France

**Keywords:** Case management team, Multidisciplinary, Case managers, Integration

## Abstract

**Background:**

The case management process is now well defined, and teams of case managers have been implemented in integrated services delivery. However, little is known about the role played by the team of case managers and the value in having multidisciplinary case management teams. The objectives were to develop a fuller understanding of the role played by the case manager team and identify the value of inter-professional collaboration in multidisciplinary teams during the implementation of an innovative integrated service in France.

**Methods:**

We conducted a qualitative study with focus groups comprising 14 multidisciplinary teams for a total of 59 case managers, six months after their recruitment to the MAIA program (*Maison Autonomie Integration Alzheimer*).

**Results:**

Most of the case managers saw themselves as being part of a team of case managers (91.5%). Case management teams help case managers develop a comprehensive understanding of the integration concept, meet the complex needs of elderly people and change their professional practices. Multidisciplinary case management teams add value by helping case managers move from theory to practice, by encouraging them develop a comprehensive clinical vision, and by initiating the interdisciplinary approach.

**Conclusions:**

The multidisciplinary team of case managers is central to the implementation of case management and helps case managers develop their new role and a core inter-professional competency.

## Background

The health system in France, as in most industrialized countries, is described as fragmented and more centered on managing acute diseases than chronic health conditions [1,2]. In response to these dysfunctions, international experiments have integrated support and care services, with positive impacts on health parameters and resource use in elderly populations [3,4]. According to Leutz, integration is defined as “the search to connect the health care system (acute, primary medical, and skilled) with other human service systems (long-term care, educational and vocational and housing services) to improve outcomes (clinical satisfaction and efficiency)” [5]. Implementing service integration requires the involvement of a wide range of professionals, in areas from clinical practice to governance, and strong leadership is essential [6]. Among the various components of integration, case management is the mechanism that ensures successful integration of services at the clinical level for elderly people with chronic health conditions [7].

Case management is a well-defined process that includes the identification of a population of elderly people with complex health problems, an exhaustive needs assessment, the planning of services, follow-ups and re-assessments in the home [8]. Case management is a new function in France, exercised by case managers who are not physicians. In so-called intensive case management, the case manager takes responsibility for the entire process for a case load of no more than 40 persons [9]. At the clinical level, the case manager coordinates all healthcare professionals in order to meet the needs of elderly persons [10]. Implementation of case management is generally accompanied by establishing a team of case managers in each service area [11,12]. These case managers may be mono-disciplinary and are often nurses, but there may also be multi-disciplinary teams (e.g. nurses, social workers, psychologists, occupational therapists). Although clinical collaboration between case managers and healthcare professionals has been widely studied in the integrated services models implemented in various countries [13-15], we know little about the collaboration that develops between case managers within case management teams. Furthermore, to our knowledge no study has examined whether there is value in having multidisciplinary case management teams.

In France, wide-scale implementation of integrated services began in 2009 with the implementation of the government’s Alzheimer plan and the establishment of the MAIA program (*Maisons Autonomie Intégration Alzheimer*, or homes for the autonomy and integration of Alzheimer patients) [16] following encouraging results in local initiatives [17,18]. To achieve their integration goal, MAIAs include a case management component that was implemented in 15 experimental pilot projects around the country. These newly recruited case managers perform intensive case management for elderly persons living at home with complex health conditions.

This study’s objectives are to develop a fuller understanding of the role played by the case manager team and identify the value of inter-professional collaboration in multidisciplinary teams during the implementation of the MAIA program. Interactions between case managers with different educational backgrounds may play a critical role by enhancing the interdisciplinary process. The French setting offers an exceptional opportunity to study an implementation of multidisciplinary teams of these new professionals.

## Methods

We conducted focus groups to investigate the role played by multidisciplinary teams of case managers as well as inter-professional collaboration factors. Given the lack of knowledge about teams of case managers, a qualitative method was deemed appropriate, and we used a grounded theory approach to analyze the data [19].

### Description of MAIAs

France’s third Alzheimer plan is a presidential mandate priority focused on persons and their caregivers. It favours home care and quality in long-term care facilities. Services are integrated under a program called *Maisons pour l*’*Autonomie et l*’*Intégration des Malades Alzheimer* (MAIA, or homes for the autonomy and integration of Alzheimer patients) [16]. These integrative units are based on the six components of integration in the Program of Research on Integration for Maintenance of Autonomy (PRISMA) initially established in Quebec [20] and then in France [17]. The six components comprise mechanisms for joint action at all decision-making levels, the reorganization of access to services through a single integrated point, the assignment of case managers to persons in complex situations, the introduction of a standardized multidimensional evaluation tool, the application of personalized service plans and the implementation of an information sharing system [21,22].

At the end of 2008, 15 sites were selected from a list of 100 potential sites. A local change-management agent was recruited to develop the service area partnership, and case managers were recruited one year later. Most of the case managers were recruited on a contractual basis with contracts of specified terms, and the others were provided by partners. Each site had one team of case managers, and the size of each team was based on the elderly population served. Two to eight case managers were hired per experimental site to manage cases on either a full-time or part-time basis. At the beginning of their practices, all of the case managers had to follow the same training on basic case management principles (120 hours over 6 months). The sites had chosen evaluation tools for use by case managers from three tools: GEVA-A (*Guide d*’*EVAluation des besoins des personnes Agées* [guide for evaluating the needs of elderly persons]) developed in France; OEMD-SMAF (*Outil d*’*Evaluation MultiDimensionel basé sur le Système de Mesure de l*’*Autonomie Fonctionnelle* [a multidimensional evaluation tool based on the system for measuring functional autonomy]) [23]; and RAI-HC (Resident Assessment Instrument-Home Care) [24].

### Study population

The study population was recruited in 2010 from the 14 sites with teams of case managers. One site had only one case manager, and this site was excluded because the case manager was newly recruited and lacked experience as a case manager. All the teams were multidisciplinary, with at least one nurse at 14 sites, one social worker at 12 sites, one psychologist at 6 sites and one occupational therapist at 4 sites. Three case managers had legal backgrounds and were classified in the “other” group. The case managers worked for at least six months following their recruitment. Whatever their initial backgrounds, all the case managers assumed only the role of case manager at their site.

All the case managers were approached to participate in the study. One case manager refused to participate and five were not available on the day that the data was collected. No differences were found between the profile of our respondents and the profile of those who declined to be interviewed. A total of 59 case managers at 14 sites participated in the focus groups. Among them, 24 were social workers (40.7%), 15 were nurses (27.1%), 11 were psychologists (18.6%), 5 were occupational therapists (8.4%) and 3 came from other professions (5.2%). The number of case managers per site varied from two to eight. Most of them worked full time as case managers (74.6%).

The study was approved by the Ambroise Paré Hospital research review board. Informed written consent was obtained from all participants.

### Data collection

Qualitative data were collected at each site by holding focus groups. Focus groups served as our main source of data. One focus group was conducted per site, with participation by the entire team of case managers working at this site. The decision was made to form focus groups with all the professional case managers, independent of their original professions. The focus groups were held at each site in a quiet space where the case managers practised. All the focus groups were conducted by the same person, a public health physician who was trained in qualitative methods. The focus groups lasted between 120 and 180 minutes.

An interview guide was developed based on the current literature on case management. The literature helped us address unanswered questions concerning the role played by a team of case managers. The resulting interview guide was refined in the field following an initial pilot interview. The interview guide began with a very general question on professional practices and then moved on to more specific questions designed to explore the implementation of case management: (1) participants’ motivations for becoming a case manager, (2) the activities of case managers, (3) the team of case managers and partnering, (4) the implementation of case management (see Additional file 1).

A short anonymous questionnaire was administered following the focus group. It provided socio-demographic data and information on individual perceptions of the multidisciplinary team of case managers.

To complement the focus group, two researchers relied on non-participant observations and spent time at various community-based health services in order to observe key professionals’ practices and information exchange. The documentation collected at each site included annual reports, minutes, and the booklets given to the users in order to get a better understanding of the organizational structure of the teams and their missions.

### Coding and analysis

Transcripts were produced, read and coded by two of the researchers. The coding was validated by another researcher, who used a consensus approach to resolve discrepancies [25]. The coding and analysis was performed using the Strauss and Corbin grounded theory approach, consisting of open, axial and selective coding [26] in order to identify relevant categories and relationships. Nvivo8 software was used to code the data and a standardized method of qualitative analysis was applied. We began with a round of open coding of the interviews focused on issues pertaining to relationships. The codes were descriptive, based on words used by participants such as “colleague,” “background,” and “difficulties.” Then, following the application of an axial coding strategy [26], codes with the same content and meaning were grouped into categories (e.g. the missions of case managers, collaboration, changes to practices). Following selective coding, the identified categories were further defined, developed, and refined and brought together in order to tell a larger story (e.g. a strong sense of belonging to a team, meeting the complex needs of elderly people through a comprehensive clinical vision). The analytical process was repeated until saturation, i.e. the point at which additional analysis repeatedly confirms previously made interpretations. The research team validated and discussed the final coding scheme and analysis to ensure that the results were not biased by spurious association. Analysis of observations and documentation were used to validate the information obtained from the interviews.

## Results

We found a strong sense of belonging to a team among the case managers. Then we identified the role played by the team of case managers and the value in having multidisciplinary case management teams under three emergent themes: having a comprehensive understanding of integration concepts within a practical application, developing a comprehensive clinical vision in order to meet the complex needs of elderly people, and affecting change in professional practices under an interdisciplinary approach.

### Strong sense of belonging to a team

Results from the questionnaire showed that the case managers had a strong perception of belonging to a team (91.5%); furthermore, 55.9% answered “yes, often” and 35.6% answered “yes, always.” The proportion was higher among the psychologists, occupational therapists and the “other” group, with 100% answering that they belonged to a team, followed by 93.8% of nurses and 83.3% of social workers (see Table 1).

**Table 1:** Description of the sample and case managers’ perceptions of belonging to a team

	**Total**	**Social workers**	**Nurses**	**Psychologists**	**Occupational therapists**	**Other**
N (%)	59	24 (40.7)	16 (27.1)	11 (18.6)	5 (8.4)	3 (5.2)
Full-time (Yes, %)	74.6	87.5	68.8	54.5	60	100
Perception of being part of a team (Yes, %)	91.5	83.3	93.8	100	100	100

### Comprehensive understanding of integration concepts within a practical application

Most of the case managers found that developing a comprehensive understanding of integration concepts is difficult and takes time. Moreover, they felt that integration implies a radical change in how services and professional practices are organized.

“*I find it difficult. It took some time for me to*…*how can I say it*…*develop a comprehensive view of the concept because*, *you know*, *it revolutionizes our approach to work*; *that*’*s it. It isn*’*t that you work with someone from time to time*, *talk about things. It*’*s really a fundamental change*, *you know*, *and it takes time to get it going*, *really it*’*s a question of*… *It*’*s very long*, *you know*.” (*CM*, *Site E*)

Perceptions about integration change over time as the case managers interact, and improving their understanding is a collective undertaking. In the multidisciplinary teams, they could translate their understanding of the integration concept into concrete practices.

“*I realized that my colleagues had the same vague ideas*, *but by sharing*, *things became a bit clearer*, *at least things became more meaningful in light of what others had to say. Finally our questions led to something concrete*, *and afterwards*, *we could see things differently*.” (*CM, Site L)*

The case managers expressed doubts about case management missions and, in particular, about the extent of their interventions. For them, the crux of the problem lies in the case manager finding his or her place in the support provided to the elderly. Case managers counted on their colleagues in the multidisciplinary team to clarify the limits of their missions.

“As for the case management missions, we often talk to each other. Sometimes I have my doubts, I’m not yet sure about everything, how far our support should go… There have been times when I’ve wondered, is this or that part of my mission, to physically support people who are consulting, for example, or am I doing too much, and we talk about it.” (CM, Site A)

The case managers believed that they would not be able to easily discuss patients’ needs and care goals with healthcare professionals by using the assessment and planning tools. In the multidisciplinary team, case managers learned together how to use the tools, and they tested their new positions before sharing information with healthcare professionals.

“The idea is that we’ll be able to use the tools to present our situations to the team and to those of us who know a bit more about them, even if they haven’t mastered them yet, in order to have a chance to use them and get very comfortable using them in the team, so that we can then communicate with partners.” (CM, Site L)

### Obtain a comprehensive clinical vision in order to meet the complex needs of elderly people

For case managers, the elderly people receiving their care represent complex cases. There are many criteria for complexity, including a mix of medical, psychological and social problems, and this makes home support difficult.

“For me, there are several complexity criteria. Usually the elderly person is in denial, and this can foil the efforts of most professionals. It’s a precarious social situation: the person is isolated, sometimes without a caregiver but with a life plan that includes staying in their home. Sometimes the complexity may be related to the sheer number of professionals involved, but each professional is working on their own and it’s very difficult to coordinate everything, what with the family conflicts and exhausted caregivers.” (CM, Site N)

For the case managers, case management would not be possible without the day-to-day presence of other case managers.

“As a case manager, I can’t imagine managing cases on my own or as part of a smaller team. Case management is teamwork.” (CM, Site L)

Case managers need technical support in their clinical interventions. Within their multidisciplinary teams, they bring different competencies to bear when addressing a specific health problem.

“Based on the original training, I must be honest, I’ll call Y because she’s trained as a nurse and, for an occasional need for information, I need her view. Or I’ll call X (a psychologist), who’ll help me with everything related to cognitive development.” (CM, Site D)

All the case managers spoke of the richness of the multidisciplinary dimension of their teamwork, which allows them to share differing points of view on how to follow up in specific situations. This sharing helps them acquire a comprehensive vision of the needs of the elderly.

“Yes, what’s so rewarding is our different educational backgrounds… We realize that each of us has his or her own vision of things, and it’s interesting to have these different points of view. For sure it’s useful. Yes, I find it very valuable. The fact that we’re a group with members from different professions, and that this makes us look far beyond our borders.” (CM, Site L)

The case managers said that it is often psychologically demanding to monitor an elderly population using case management. Working in a multidisciplinary team allows them to talk about problems, express doubts, put things in perspective and find the confidence they need to deal with certain situations.

“In the team, when we have a question and aren’t sure quite what to do, when we need input from almost everyone. When we have doubts, but when we’re fed up, too. When we’ve reached a dead end and no longer know what to do. It’s good to share your concerns, your doubts. It helps you make decisions.” (CM, Site H)

Most of the case managers insisted on the need for continuity of care to improve the quality of their interventions and meet people’s needs. At almost all the sites the case managers had organized themselves within the multidisciplinary teams so that case management would continue even if a case manager was absent.

“In each situation, there’s always an owner and an alternate. We decided that, in fact: in every situation there would always be two of us. If the owner goes on leave, then the alternate takes over.” (CM, Site H)

### Changes in professional practices under an interdisciplinary approach

Case managers said that they felt better understood by the other case managers than they did by other healthcare professionals. Within the multidisciplinary team, the case managers shared a vision and intervention objectives when dealing with their elderly population.

"For me, I feel more easily heard and understood by my fellow case managers because we try to take the same approach (compared to other professionals)… a shared philosophy… a similar thought process, despite all our differences. I think that we all think the same way. We may differ on some points, but overall… We try to be positive, have the same goals.” (CM, Site B)

The case managers pointed out how, when the case management was initially being implemented, each of them had different practices based on their professional backgrounds. Collaborating with each other had the effect of gradually standardizing day-to-day practices.

“At first I found that we had very different ways of approaching situations, considerable differences in how we understood things. Depending on whom you spoke to, people saw things very differently. I noticed that my colleague X had a very specific way to begin managing situations, and that my approach wasn’t exactly the same as colleague Y’s approach. With time, I’d say that the team began to be more homogeneous.” (CM, Site C)

The case managers stressed the need to avoid situations where case managers use different practices in their collaboration with healthcare professionals. This allows partners to make case management more understandable, which is essential to implementing case management in a given service area.

“Concerning the partners we work with, I think that for them it’s important to avoid situations where one partner can say that it works this way with the case manager in this sector, but partners in another sector describe something different. I think that eliminating disparities between case managers encourages effective case management.” (CN, Site D)

Even if the practices of case managers were becoming standardized and the approach was becoming more interdisciplinary, the influence of professionals’ initial training did not disappear completely. Some case managers rely on their initial training when taking up case management. For most case managers, their initial training tends to be more apparent, particularly during the meetings of multidisciplinary teams.

“No, for me, I haven’t completely left my profession because I use what I did before and rely on it to become a case manager.” (CM, Site A)

“My profession is still there, in the wings. Above all, it’s present in the teamwork.” (CM, Site G)

## Discussion

The multidisciplinary case management team appears to play a key leverage role when case management is being implemented. Teams help case managers develop a comprehensive understanding of the integration concept, meet the complex needs of elderly people and change their professional practices. Multidisciplinary case management teams add value by helping case managers move from theory to practice, by encouraging them to develop a comprehensive clinical vision, and by initiating the interdisciplinary approach.

Case management is a process that takes place within an integration mechanism with different components, not outside of it [27]. The mechanism is complex, and our results confirm this fact, as case managers need time to absorb how the full mechanism works. It has been shown that training case managers on integration concepts is important [20]. The case managers in the MAIA program were all trained, but our results suggest that training is not enough to fully adjust to practice within this new competency. Our study shows that a team of case managers represents a forum for group learning, so that the professionals’ knowledge of integration components can improve throughout the implementation of this form of organization. Furthermore, the function of case manager leads to changes in the collaboration process with healthcare professionals. In clinical practice, case managers become responsible for coordination of care and must know how to delegate to partners [28]. Our results show that the case manager’s missions and, in particular, the limits of his or her interventions are not always clear and must be determined on a case-by-case basis. The case managers discuss changes in their situations at meetings of the multidisciplinary team so that, together, the professionals can adjust and validate their interventions with other healthcare professionals. There is an international consensus that case managers’ use of tools represents an essential activity that affects the effectiveness of their actions [29]. It has been shown that use of these tools in the case management implementation phase is associated with the emergence of a new professional identity [30]. In the MAIA program, all the case managers had received classroom training on how to use these tools, and our results show that they needed to improve their skills in the practical application of these tools. This is why the case managers were relying on the multidisciplinary team to learn how to make practical use of these tools before sharing information with healthcare professionals.

The complex situations experienced by elderly persons are marked by instability, uncertainty and unpredictability [31], with disabilities and a high risk of death [32]. Our results confirm that elderly people present complex situations and that case managers need strong support in their clinical interventions. We have shown that the multidisciplinary team provides case managers with multidimensional technical advice and support in their decision making as they monitor the elderly over the long term. Working in a multidisciplinary team of case managers leads to the acquisition of a comprehensive and shared vision of the problems faced by the elderly as different competencies are brought to bear. It has been shown that the presence of different educational backgrounds stimulates interaction between case managers, and that having a comprehensive vision of needs serves as a link between service evaluation and service planning [33]. Furthermore, the coordination function has been described as difficult because the health care professionals are not accustomed to working together [34] and the care processes are neither standardized nor routine [35]. Case managers have often been described as acting as integration “peacekeepers” by coordinating the professionals’ contributions and giving them a way to understand the actions that are required. This is why the case manager function is difficult psychologically, and our results show that case managers need to have a place where they can talk about their problems and find support. The multidisciplinary team is a vessel in which case managers can talk about what makes them uneasy, express their doubts and find encouragement for overcoming their problems. It has been shown that case managers need support because they sometimes feel isolated [36] in a context where they must [37] improve the quality of homecare [38] and limit unmet needs [34,39].

Case managers are receptive to the concept of case management but are still ambivalent about standardizing their practices [40]. Case managers are accountable for systematically performing case management tasks and therefore must ensure that these practices are standardized with those of their case manager colleagues. Our results show that it is through their work in a multidisciplinary team that the practices used by professionals with different initial training are standardized and interdisciplinary practice is initiated.

First, our results show that case managers feel understood in their multidisciplinary team, share a common set of values and become aware of the need to standardize their professional practices. An interdisciplinary approach is constructed among the different professionals involved, and it has been shown that the situation is more favourable to this construction if the professionals complement each other rather than compete with each other [41]. Second, our results underscore how case managers want to have standardized practices in order to make their new function easy for healthcare professionals to grasp. Such practices serve to clarify and legitimize the case manager’s role, and this supports working in partnership to build a clinical consensus. Indeed, the healthcare professionals are involved in the case management process and also help build integration [42]. Establishing the case management competency does not, however, totally eliminate the role played by case managers’ initial backgrounds. Our results show that this new professional identity is related to the case manager’s initial background, but the links between the two are not always clear during the transition. The fact that our study was conducted in the implementation phase may explain part of this confusion. Additional work is needed conducted after the implementation phase in order to study the relationships between these overlapping professional identities.

Even though we used a large sample of case managers, the qualitative data from the focus groups does not allow us to identify potential differences in perceptions of the multidisciplinary team’s role based on a case manager’s initial background. Furthermore, the teams of case managers varied in size from one site to another, and this may have influenced the participants’ answers. But the larger teams often worked at several sites, which may have limited this bias. Finally, the MAIA sites were not always comparable in terms of the degree to which they had implemented the different components of integration, and this may have influenced the case managers’ experience with the multidisciplinary team dimension.

## Conclusion

Implementing case management is accompanied by a profound change in professional practices and needs to occur in a supportive environment. Integration helps case managers reduce fragmentation between services, and the multidisciplinary team allows them to build their new competency. This is why case management cannot be applied outside of integrated services delivery, and case management cannot be implemented without having case managers participate in a multidisciplinary team. Future research should examine the role of the case manager team sometime after the case management implementation phase and in terms of the case managers’ initial training.

## Competing interests

The authors declare that they have no competing interest.

## Authors’ contributions

MDS, IV and DS designed the study. MDS and DS developed the structured interviews. MDS conducted the interviews. MDS, HT and DS analyzed all the interviews. All the authors read and approved the final manuscript.

## Pre-publication history

The pre-publication history for this paper can be accessed here:

http://www.biomedcentral.com/1472-6963/14/159/prepub

## Supplementary Material

Additional file 1 Interview Guide. Click here for file
