# Supplementary material for: Multidisciplinary teams of case managers in the implementation of an innovative integrated services delivery for the elderly in France
Source: BMC Health Serv Res. 2014 Apr 7;14:159. doi: 10.1186/1472-6963-14-159 (PMC4021253; doi:10.1186/1472-6963-14-159)
Supplement: Additional file 1 — Interview Guide. [file 1472-6963-14-159-S1.doc]

**Interview Guide**

***Theme 1: Motivation behind becoming a case manager***

Do you remember the first time you heard about MAIAs and case management?

What did you think about it? What do you think about it today?

How do you associate the MAIA program with case management?

***Theme 2: The case management activity***

How do you present yourself to someone in a complex situation?

How often do you see your clients, on average?

Tell us about your relationships with family caregivers.

Tell us about your relationships with the professionals working in social or medical-social areas.

Tell us about your relationships with hospital professionals.

What form does your intervention take when the person you are following is hospitalized?

Tell us about your relationships with attending physicians.

What means of communication do you use most?

Under what conditions do you think the case manager should be able to use an interdisciplinary meeting?

***Theme 3: Relationships within the team and with partners***

What do you think of your relationship inside the team of case managers?

What do you think of your relationship with the leader? What do you think of your relationship with the sponsor of the MAIA project?

What roles do the leader and sponsor play in your case management activities?

How, amongst yourselves, do you discuss the situations you are monitoring?

***Theme 4: Degree to which case management has been implemented***

To what extent would you say that case management has been implemented in your MAIA service area?

What needs to be added to achieve total implementation?

Have you ever had trouble with a partner in an intervention because you didn’t feel that your role was fully legitimized?

What kind of effort do you feel the partners are making to ensure that your intervention manages to reduce assessment duplications? Can you provide concrete examples?

What allows you to ensure that everyone in your service area who should have case management is calling on your services?
